# Epidemiology of sports-related concussion in seven US high school and collegiate sports

**DOI:** 10.1186/s40621-015-0045-4

**Published:** 2015-06-16

**Authors:** Stephen W Marshall, Kevin M Guskiewicz, Viswanathan Shankar, Michael McCrea, Robert C Cantu

**Affiliations:** 1 Department of Epidemiology, University of North Carolina at Chapel Hill, Chapel Hill, NC USA; 2 Department of Exercise and Sport Science, University of North Carolina at Chapel Hill, Chapel Hill, NC USA; 3 Matthew Gfeller Sport-Related Traumatic Brain Injury Center, University of North Carolina at Chapel Hill, Chapel Hill, NC USA; 4 Injury Prevention Research Center, University of North Carolina at Chapel Hill, Chapel Hill, NC USA; 5 Department of Epidemiology & Population Health, Albert Einstein College of Medicine, Bronx, NY USA; 6 Neuroscience Center, Waukesha Memorial Hospital, Waukesha, WI USA; 7 Department of Neurology, Medical College of Wisconsin, Milwaukee, WI USA; 8 Neurosurgery Service, Emerson Hospital, Concord, MA USA

**Keywords:** Traumatic brain injury, Head injury, Football, Lacrosse, Soccer

## Abstract

**Background:**

The epidemiology of sports-related concussion is not well-described in the literature. This paper presents a descriptive epidemiology of concussion in seven high school and collegiate sports.

**Methods:**

We used the data from *Concussion Prevention Initiative* (CPI), which enrolled 8905 athletes at 210 high schools and 26 colleges in a prospective cohort study of 7 sports (football, men’s and women’s soccer, men’s and women’s lacrosse, and men’s and women’s ice hockey) between 1999 and 2001. Injury risks and injury rates were used to characterize the incidence of concussion, and changes in symptoms over time were described.

**Results:**

A total of 375 concussions were observed. The incidence of concussion was highest in football, followed by women’s lacrosse, men’s lacrosse, men’s soccer, and women’s soccer (only 10 ice hockey teams were included, too few to quantify incidence). The rate of incident concussion was strongly associated with history of concussion in the previous 24 months (rate ratio = 5.5; 95 %CI: 3.9, 7.8, for 2 or more concussions relative to no previous concussion). The most common symptoms at time of injury were headache (87 %), balance problems/dizziness (77 %), and feeling “in a fog” (62 %). Loss of consciousness and amnesia were present in relatively few cases (9 and 30 %). The most common mechanism of injury was collision with another player.

**Conclusions:**

Sports-related concussions present with a diverse range of symptoms and are associated with previous concussion history.

## Background

Concussion, also known as mild traumatic brain injury, is characterized by an impact (or other application of force) to the head that triggers a disruption of normal brain function (Nilsson and Ponten 1977; Nilsson et al. 1977; Giza and Hovda 2001; Shaw 2002). The signs and symptoms of the injury are very subjective and may go unrecognized or unreported (McCrea et al. 2004). They can range from (in the most severe cases) impairments in cognitive functioning that may last for months through to (in the less severe and more typical cases) headaches, concentration problems, and dizziness that resolves within a week (Guskiewicz et al. 2004).

There is no direct therapeutic treatment for concussion. Typically, given adequate rest, the brain will reinstate normal functioning within 7–10 days following the concussive episode. The exact recovery mechanism remains unknown. Therefore, treatment for concussion essentially consists of a period of inactivity to minimize the probability a second impact will occur during the healing period. For a concussed athlete, this typically means removing the athlete from competition until the clinician determines that his/her symptoms have resolved.

Sports-related concussions result in over 200,000 emergency department visits annually (Centers for Disease Control 2007). However, because the majority of concussions are treated in settings other than an emergency department, the annual number of sports-related concussions is much higher—possibly up to 3.8 million (Langlois et al. 2006). However, the incidence and nature of sports-related concussion is poorly described in the literature (Centers for Disease Control 2007; Thurman et al. 1998). This lack of information hampers the identification and management of this injury (Collins et al. 1999). The purpose of this paper was to present a descriptive epidemiology of concussion in a variety of high school and collegiate sports.

## Methods

### Study design

The data reported here come from the *Concussion Prevention Initiative* (CPI), a randomized controlled trial of four clinical assessment protocols for managing concussion. The outcome examined in the trial was time to return to full participation in sports following concussion. In the interests of ensuring a balanced trial, all four treatment arms tracked covariate data on symptomology and recovery following concussion using standardized forms. This paper reports these covariate measures, along with the data on observed concussion incidence. As a working assumption, the measures of incidence and symptomology reported here were assumed to be independent of study arm, since the randomized study arms compared four concussion management protocols and therefore should influence neither incidence nor symptomology. Therefore, data from the four arms were pooled in all analyses reported here.

CPI prospectively enrolled a total of 8905 athletes (7513 high school athletes at 210 high schools and 1392 collegiate athletes at 26 colleges). To be eligible for the study, a school or college had to have one or more Certified Athletic Trainers (ATs) on staff.

The study included seven sports: football, men’s and women’s soccer, men’s and women’s lacrosse, and men’s and women’s ice hockey. No more than three sports were included per high school or college in order to minimize the burden of the study on each institution. Men’s and women’s lacrosse, and men’s and women’s ice hockey, are less commonly offered by schools and colleges than other sports studies. Therefore, these sports were preferentially selected at the schools and colleges that offered these sports. Schools and colleges were selected from the records of the National Athletic Trainer’s Association and through personal contacts. To minimize travel costs, all schools and colleges were located in the eastern United States (Pennsylvania, Virginia, District of Columbia, North Carolina, and South Carolina).

To be eligible for the study, an athlete had to play in at least one of the study sports for his or her institution and speak English. We attempted to enroll every athlete on every eligible team. The study was approved by the Institutional Review Board at UNC-Chapel Hill.

### Injury definition

A concussion was defined as an injury resulting from a blow to the head which caused an alteration in mental status resulting in one or more somatic (headache, balance problems or dizziness, nausea, blurred vision, numbness/tingling, vomiting, drowsiness, fatigue, sensitivity to light, sensitivity to noise), neurobehavioral (sleeping more than usual, trouble sleeping, irritability, sadness), or cognitive (feeling “in a fog,” difficulty concentrating, difficulty remembering) symptoms (Kelly and Rosenberg 1998; Piland et al. 2003; Piland et al. 2006). Any player who, based on clinical impression, was suspected of having suffered a concussion of any severity level, and on whom a routine clinical examination for concussion or head injury would normally have been conducted prior to implementation of this research protocol, was included in this study. A concussion was excluded if there was evidence of associated drug use or concurrent non-concussive injuries (such as fractured bones) or if the concussion was not confirmed by clinical assessment. The AT at each school or college was responsible for identification and confirmation of concussions.

### Data collection

At study baseline, all schools and colleges participating in this study were visited by an investigator and/or staff member from the research team. The purpose of the visit was to provide training in the study protocol (including the above injury definition) and assist with baseline data collection.

All athletes on a team who consented were tested at baseline. Each athlete was administered a baseline questionnaire, which included questions on all previous concussions, and the timing of these concussions, for a period of up to 7 years in the past.

Athletes were followed prospectively for concussions through their playing career at each institution. Once a concussion was identified, the AT at the school or college used a standardized graded symptom checklist to assess the symptomology at time of injury, 3 h post-competition and 1, 2, 3, 5, and 7 days post-injury. Each symptom was scored “0” if absent or, if present, given a rating between 1 (mild) and 6 (severe). If the athlete was unavailable for testing on the designated day, the AT collected the data on the closest possible day. The AT also used a standardized form (the “concussion index”) to record details about each injury (mechanism of injury, loss of consciousness, presence of amnesia, etc.). At four schools, the AT was unable to fully comply with the protocol, resulting in incomplete data (this data was excluded from analysis).

### Statistical analysis

Injury risks and injury rates were used to characterize the incidence of concussion (Knowles et al. 2006). Injury risk is an incidence proportion (Rothman and Greenland 1998), defined as the number of concussed athletes divided by the total number of athlete-seasons. This measure of incidence has a natural interpretation as the average probability that an athlete will be concussed during a season and therefore is meaningful for clinicians, athletes, parents, and coaches. Injury rate is an incidence rate (Rothman and Greenland 1998), defined as the number of concussions divided by the total number of “athlete-exposures” that occurred during the follow-up period. Note that the number of concussions will be greater than the number of concussed athletes if there were multiple concussions to the same athlete in the same season. Athlete-exposure is based on the concept of person-time at risk. It quantifies the total number of participant episodes at risk. For example, a team of 10 athletes who participated in 5 games and 50 team practices would accumulate 50 athlete games and 500 athlete practices for a total of 550 athlete-exposures. Injury rate is a measure of incidence which facilitates scientific comparisons of the effect of various factors, such as playing position or injury history.

Compiling a complete census of athlete-exposures in 8905 athletes would have been a labor-intensive task beyond the resources of this study. The total number of athlete-exposures was therefore estimated using data from a detailed exposure survey compiled by a convenience sample of nine high schools and six colleges. The detailed exposure survey collected data on the total number of pre-season, regular-season, and post-season exposures (both games and practices) for every athlete (*n* = 892) in the study at the 15 selected schools. We used these data to compute the average number of athlete exposures in each cell of a four-way table comprising sport by gender by academic year (freshman, sophomore, junior, senior) by setting (high school or college). These averages were assumed to be representative of the entire population and were applied to each of the 8905 athletes in the main cohort; however, representativeness cannot be guaranteed. Standard formulae were used for the confidence intervals, under the assumption that sampling the denominator counts had minimal effect on the rate variance.

There is no clear consensus on the optimal way to group symptoms for data analysis, so clusters suggested by factor analysis of baseline (pre-injury) data were used (Piland et al. 2003; Piland et al. 2006) with one modification: we classified drowsiness and fatigue as somatic symptoms.

## Results

Average follow-up was 2.2 seasons and 161 athlete-exposures per athlete. The study followed an approximately equal number of football, men’s soccer, and women’s soccer teams—approximately 100 in each sport—but the number of athletes followed was much greater in football than any other sport, due to the larger team size in football (Table 1).

**Table 1:** Number of schools and athletes enrolled, by sport

	High school (*n* = 210)			College (*n* = 26)		
	No. teams	No. athletes	No. concussions	No. teams	No. athletes	No. concussions
Football	102	3457	197	8	716	42
Men’s soccer	97	1649	49	8	191	5
Women’s soccer	94	1619	31	9	196	4
Men’s lacrosse	41	713	14	3	88	2
Women’s lacrosse	26	504	21	6	125	5
Men’s ice hockey	3	73	1	2	54	0
Women’s ice hockey	3	42	1	2	38	1
Total	366	7513	314	38	1393	59

### Incidence

A total of 375 concussions were observed. The average overall incidence rate was 26.1 per 100,000 athlete-exposures (95 %CI: 23.5, 28.7; confidence limit ratio (CLR): 1.2), and the overall risk for an average season was 1.8 per 100 athletes (95 %CI: 1.6, 2.0; CLR: 1.2). The incidence of concussion was highest in football, followed by women’s lacrosse, men’s lacrosse, men’s soccer, and women’s soccer (Tables 2–4). There were too few ice hockey teams to quantify incidence (only three ice hockey concussions were observed).

**Table 2:** Incidence of concussion in football

	No. (%) of concussions	Injury risk (incidence proportion) per season^a^				Injury rate (incidence rate) per 100,000 athlete-exposures			
		Athlete-seasons	Risk (%)^a^	95 %CI	CLR	Athlete-exposures	Rate	95 %CI	CLR
Overall	239 (100)	8524	2.6	2.3, 3.0	1.3	676,274	35.3	30.9, 39.8	1.3
Exposure type									
Games	148 (61.9)	––	––	––	––	77,767	190.3	159.7, 221.0	1.4
Practices	85 (35.6)	––	––	––	––	598,507	14.2	11.2, 17.2	1.5
Setting									
High school	197 (82.4)	7247	2.6	2.2, 3.0	1.4	566,063	34.8	29.9, 39.7	1.3
College	42 (17.6)	1277	2.9	2.0, 3.8	1.9	110,212	38.1	26.6, 49.6	1.9
Position^b^									
Defensive back	23 (9.6)	811	2.7	1.6, 3.8	2.4	65,327	35.2	20.8, 49.6	2.4
Defensive lineman	26 (10.9)	1090	2.3	1.4, 3.2	2.3	87,072	29.9	18.4, 41.3	2.3
Linebacker	29 (12.1)	909	2.8	1.7, 3.8	2.2	72,539	40.0	25.4, 54.5	2.1
Offensive lineman	48 (20)	2051	2.2	1.6, 2.9	1.8	161,901	29.7	21.3, 38.0	1.8
Quarterback	19 (7.9)	506	3.6	1.9, 5.2	2.7	39,737	47.8	26.3, 69.3	2.6
Receiver	31 (13)	1264	2.4	1.5, 3.2	2.1	100,046	31.0	20.1, 41.9	2.1
Running back	40 (16.7)	1135	3.3	2.2, 4.3	2.0	89,028	44.9	31.0, 58.9	1.9
Special teams and kicker	5 (2.1)	230	2.2	0.3, 4.1	13.7	18,826	26.6	3.3, 49.8	15.2
Tight end	13 (5.4)	487	2.7	1.2, 4.1	3.4	38,488	33.8	15.4, 52.1	3.4

*CI* confidence interval, *CLR* confidence limit ratio

^a^Injury risk is the average probability that an athlete will be concussed during a season

^b^There were six concussions (2 %) in activities other than games and practices; position is undefined for these injuries

**Table 3:** Incidence of concussion in men’s and women’s soccer

	No. (%) of concussions	Injury risk (incidence proportion) per season^a^				Injury rate (incidence rate) per 100,000 athlete-exposures			
		Athlete-seasons	Risk (%)^a^	95 %CI	CLR	Athlete-exposures	Rate	95 %CI	CLR
Men’s soccer	54 (100)	4029	1.3	0.9, 1.6	1.7	285,931	18.9	13.9, 24.0	1.7
Exposure type									
Games	43 (79.6)	––	––	––	––	79,890	53.8	37.7, 69.6	1.9
Practices	9 (16.7)	––	––	––	––	206,041	4.4	1.5, 7.2	4.8
Setting									
High school (*n* = 97)	49 (90.7)	6661	1.3	0.9, 1.7	1.9	257,835	19.0	13.7, 24.3	1.8
College (*n* = 8)	5 (9.3)	368	1.4	0.2, 2.5	12.5	28,097	17.8	2.2, 33.4	15.2
Position									
Goalkeeper	11 (20.4)	292	3.4	1.3, 5.5	4.2	20,717	53.1	21.7,84.5	3.9
Defense	16 (29.6)	1172	1.3	0.6, 1.9	3.2	83,279	19.2	9.8, 28.6	2.9
Midfield	13 (24.1)	1308	1.0	0.5, 1.5	3.0	92,820	14.0	6.4, 21.6	3.4
Forward	14 (25.9)	1196	1.2	0.6, 1.8	3.0	84,742	16.5	7.9, 25.2	3.2
Women’s soccer	35 (100)	3798	0.9	0.6, 1.2	2.0	259,678	13.5	9.0, 17.9	2.0
Exposure type									
Games	32 (91.4)	––	––	––	––	65,923	48.5	31.7, 65.4	2.1
Practices	3 (8.6)	––	––	––	––	193,755	1.6	0.0, 3.3	0
Setting									
High school (*n* = 94)	31 (88.6)	3443	0.9	0.6, 1.2	2.0	231,221	13.4	8.7, 18.1	2.1
College (*n* = 9)	4 (11.4)	355	1.1	0.03, 2.2	73	28,457	14.1	0.3, 27.8	99
Position^b^									
Goalkeeper	8 (22.9)	353	2.3	0.7, 3.8	5.4	24,301	32.9	10.1,55.7	5.5
Defense	7 (20)	1075	0.7	0.2, 1.1	5.5	73,967	9.5	2.5, 16.5	6.7
Midfield	9 (25.7)	1423	0.6	0.2, 1.0	5.0	97,019	9.3	3.2, 15.3	4.8
Forward	9 (25.7)	899	1.0	0.4, 1.7	4.3	61,095	14.7	5.1, 24.4	4.8

*CI* confidence interval, *CLR* confidence limit ratio

^a^Injury risk is the average probability that an athlete will be concussed during a season

^b^For women’s soccer, there were two concussions (6 %) with missing data for position

**Table 4:** Incidence of concussion in men’s and women’s lacrosse

	No. (%) of concussions	Injury risk (incidence proportion) per season^a^				Injury rate (incidence rate) per 100,000 athlete-exposures			
		Athlete-seasons	Risk (%)^a^	95 %CI	CLR	Athlete-exposures	Rate	95 %CI	CLR
Men’s Lacrosse	16 (100)	1246	1.3	0.7, 1.9	1.7	70,341	22.8	11.6, 33.9	2.9
Exposure type^b^									
Games	10 (62.5)	––	––	––	––	17,801	56.2	21.4, 91.0	4.3
Practices	4 (25)	––	––	––	––	52,540	7.6	0.2, 15.1	99
Setting									
High school (*n* = 41)	14 (87.5)	1033	1.4	0.7, 2.1	3.2	57,054	24.5	11.7, 37.4	3.2
College (*n* = 3)	2 (12.5)	213	0.9	0.3, 2.2	7.3	13,287	15.1	0.0, 35.9	––
Position									
Goaltender	0 (0)	95	0.0	––	––	5366	0.0	––	––
Defense	5 (31.3)	361	1.7	0.4, 3.0	8.8	20,453	29.3	5.9, 52.8	9.0
Midfield	5 (31.3)	299	1.7	0.2, 3.1	5.0	16,986	29.4	3.6, 55.2	15.2
Attack	6 (37.5)	471	1.1	0.1, 2.0	14.2	26,420	18.9	2.3, 35.5	15.2
Women’s Lacrosse	26 (100)	1713	1.5	0.9, 2.1	2.2	111,195	23.4	14.4, 32.4	2.3
Exposure type^b^									
Games	22 (84.6)	––	––	––	––	26,448	83.2	48.4, 117.9	2.4
Practices	3 (11.5)	––	––	––	––	84,747	3.5	0.0, 7.6	---
Setting									
High school (*n* = 26)	21 (80.8)	1562	1.3	0.8, 1.9	2.5	100,098	21.0	12.0, 30.0	2.5
College (*n* = 3)	5 (19.2)	151	3.3	0.5, 6.2	13.4	11,097	45.1	5.6, 84.6	15.2
Position^c^									
Goaltender	0 (0)	103	0.0	––	––	6737	0.0	––	––
Defense	7 (26.9)	407	1.7	0.5, 3.0	6.5	26,361	26.6	6.9, 46.2	6.7
Midfield	9 (34.6)	741	1.2	0.4, 2.0	4.7	48,140	18.7	6.5, 30.9	4.8
Attack	9 (34.6)	440	2.1	0.7, 3.4	4.7	28,539	31.5	10.9, 52.1	4.8

*CI* confidence interval, *CLR* confidence limit ratio

^a^Injury risk is the average probability that an athlete will be concussed during a season

^b^Data was missing on type of exposure (game or practice) for two concussions (13 %) for men’s lacrosse

^c^There was one concussion (4 %) in activities other than games and practices for women’s lacrosse; position was undefined for this injury

Overall, nearly two thirds of concussions occurred in football (*n* = 239, 64 %). The rate of concussion in football games was approximately twice the rate in soccer and lacrosse games, and the rate of concussion in football practices was at least three times greater than the practice rate in the other sports (Tables 2–4). Overall, 71 % of concussions occurred in games and 29 % occurred in practices. The proportion of concussions that occurred in practices was 36 % for football but only 16 % for all other sports combined.

In this predominantly high school sample, quarterbacks and running backs had the highest incidence of concussion in football (Table 2). Four of the 19 injuries to quarterbacks occurred in practices (3 in high schools and 1 in college). For men’s and women’s soccer, goalkeepers had the highest incidence of concussion (Table 3). For men’s and women’s lacrosse, the incidence of concussion was lowest in goaltenders (Table 4).

### Concussion history

The association between incident concussion and a positive history of previous concussion in the past 24 months was investigated. A time-dependent concussion history variable was created so that each athlete’s concussion history could change dynamically over the follow-up period. Thus, an athlete who had 1 concussion 12 months before study baseline and had another concussion 12 months after study baseline would have a concussion history of 1 previous concussion during the first 12 months of follow-up, a concussion history of 2 previous concussions during follow-up months 13 to 24, and a concussion history of 1 previous concussion during follow-up months 25 to 36.

Treated in this time-dependent manner, a positive history of previous concussion was strongly associated with an increased rate of incident concussion (Table 5). Athletes with a history of 1 concussion in the previous 24 months had over twice the rate of concussion, compared to those with no previous concussions, and those with 2 or more previous concussions had a 5 times higher rate.

**Table 5:** History of previous concussion and incident concussion rate

Number of previous concussions within the past 24 months	No. of incident concussions	Athlete-exposures	Rate per 100,000 athlete-exposures	95 %CI	CLR	Rate ratio	95 %CI	CLR
None	247	1,228,161	20.1	17.6, 22.6	1.3	1.0	(reference)	––
1	90	174,923	51.5	40.8, 62.1	1.5	2.6	2.0, 3.3	1.6
2 or more	38	34,147	111.3	75.9, 146.7	1.9	5.5	3.9, 7.8	2.0

*CI* confidence interval, *CLR* confidence limit ratio

### Injury mechanism

For football, the most frequent injury mechanism was a collision between two players, cited as the sole mechanism in 30 % of concussions (*n* = 72) and as a contributing mechanism (i.e., occurred in combination with some mechanism) in a further 5 % (*n* = 13). Tackling an opponent, being tackled by an opponent, and blocking an opponent accounted for 20 % (*n* = 48), 14 % (*n* = 34), and 11 % (*n* = 28) of concussions as the sole mechanisms, respectively, and were contributing mechanisms in an additional 4 % (*n* = 8), 4 % (*n* = 8), and 2 % (*n* = 4), respectively.

In soccer, the mechanisms of injury were very similar for men and women. The most frequent injury mechanism was again collision between two players, the sole mechanism in 59 % (*n* = 20) of men’s soccer concussions and 36 % (*n* = 20) of women’s soccer concussions. The second most common mechanism was contact with the ball, the sole mechanism for 18 % for both men (*n* = 6) and women (*n* = 10). Although it was not important as a sole mechanism, contact with the ground was a factor in 15 % of men’s (*n* = 5) and 20 % of women’s (*n* = 11) soccer concussions.

In lacrosse, the mechanism of injury was very different between the men’s game (which permits contact) and the women’s game (which prohibits contact). The primary injury mechanism in men’s lacrosse was collision with an opponent, the sole mechanism for 69 % (*n* = 18) of concussions. For women, contact with an object (presumably the stick) predominated (sole or contributing mechanism in 50 % of cases, *n* = 8).

### Signs

A loss of consciousness was reported in only 9 % (*n* = 35) of concussions. Loss of consciousness tended to be brief (median of 5 s), and consciousness was regained in 30 s or less in 91 % of loss of consciousness cases. Only 8 of the 375 concussions reported a loss of consciousness longer than 30 s.

Amnesia was reported in 30 % of cases (*n* = 112). Anterograde (or post-traumatic) amnesia is the inability to remember the events following the injury (e.g., leaving the field of play for examination), whereas retrograde amnesia is inability to remember the events preceding the injury (e.g., the score of the game immediately prior to the injury). Anterograde amnesia without any retrograde amnesia was reported in 13 % of cases (*n* = 47), and retrograde amnesia without any anterograde amnesia was reported in 8 % of cases (*n* = 30). Nine percent of cases (*n* = 35) involved both types of amnesia.

Retrograde amnesia tended to persist for longer than anterograde amnesia. Anterograde amnesia was present for a median of 20 min, with 90 % resolving within 3 h, whereas retrograde amnesia was present for a median of 30 min, with 87 % resolving within 6 h.

### Symptoms

**Fig. 1:**
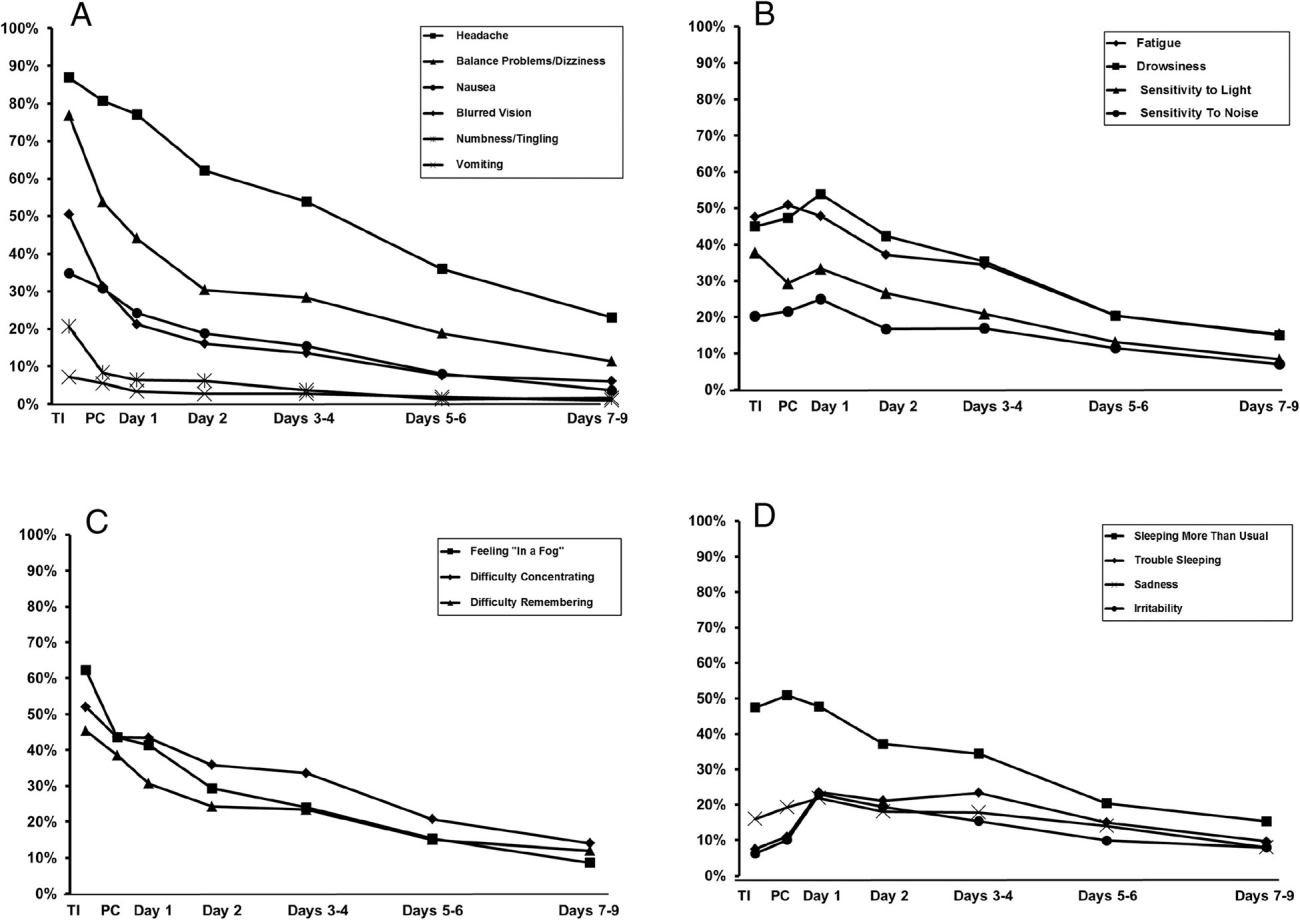
Percentage of concussed athletes reporting each symptom. **a** Somatic symptoms group 1 (monotonic decrease over time). **b** Somatic symptoms group 2 (increase to day 1, then decrease). **c** Cognitive symptoms. **d** Neurobehavioral symptoms. *TI* time of injury (*n* = 238), *PC* 3 h post-injury (*n* = 218), *Day 1* (*n* = 297), *Day 2* (*n* = 293), *Days 3–4* (*n* = 325), *Days 5–6* (*n* = 314), *Days 7–9* (*n* = 300)

Symptomology at time of injury and post-injury was recorded using the graded symptom checklist (Fig. 1, Table 6). At time of injury, the two most frequently reported symptoms were somatic: headache (87 % of subjects) and balance problems/dizziness (77 %), while the third and fourth most frequently reported symptoms were cognitive: feeling “in a fog” (62 %) and difficulty concentrating (52 %).

**Table 6:** Mean (and SD) symptom score on six-point scale^a^

	Time of injury (*n* = 238)^1^	Post-competition (*n* = 218)	Day 1 (*n* = 297)	Day 2 (*n* = 293)	Days 3–4 (*n* = 325)	Days 5–6 (*n* = 314)	Days 7–9 (*n* = 300)
Somatic group 1							
Headache	3.38 (1.42)	3.20 (1.40)	2.79 (1.38)	2.31 (1.11)	2.36 (1.30)	2.05 (1.31)	1.93 (1.23)
Balance problems/dizziness	3.07 (1.33)	2.53 (1.33)	1.99 (1.16)	1.91 (1.21)	2.10 (1.20)	1.80 (1.11)	1.71 (1.12)
Nausea	2.73 (1.33)	2.13 (1.39)	2.11 (1.27)	1.87 (1.16)	2.14 (1.21)	1.92 (1.26)	2.00 (1.41)
Blurred vision	2.76 (1.30)	2.37 (1.34)	1.97 (1.12)	1.68 (0.89)	1.80 (0.95)	1.54 (0.88)	1.28 (0.57)
Numbness/tingling	2.41 (1.41)	2.11 (1.37)	1.68 (1.1.6)	1.83 (1.15)	1.75 (1.42)	3.25 (2.06)	1.60 (0.89)
Vomiting	3.12 (1.36)	3.33 (1.87)	3.00 (0.94)	2.38 (1.06)	2.44 (1.51)	2.00 (0.63)	2.33 (2.31)
Somatic group 2							
Drowsiness	2.83 (1.36)	2.83 (1.50)	2.45 (1.32)	2.05 (1.25)	2.15 (1.31)	2.19 (1.44)	1.84 (1.24)
Fatigue	2.87 (1.50)	2.97 (1.53)	2.54 (1.35)	2.34 (1.29)	2.29 (1.37)	2.05 (1.23)	1.83 (1.35)
Sensitivity to light	2.84 (1.43)	2.70 (1.60)	2.31 (1.27)	1.82 (1.02)	1.94 (1.16)	1.71 (1.03)	1.40 (0.76)
Sensitivity to noise	2.96 (1.44)	2.74 (1.55)	2.38 (1.24)	1.78 (1.03)	1.85 (1.11)	1.64 (0.93)	1.52 (0.75)
Cognitive							
Difficulty concentrating	2.98 (1.46)	2.66 (1.45)	2.28 (1.28)	2.03 (1.20)	2.13 (1.30)	2.08 (1.34)	1.79 (1.39)
Feeling “in a fog”	2.95 (1.44)	2.74 (1.40)	2.31 (1.34)	1.92 (1.20)	2.05 (1.22)	2.00 (1.27)	1.81 (1.20)
Difficulty remembering	3.17 (1.54)	2.65 (1.54)	2.18 (1.29)	2.04 (1.25)	2.21 (1.33)	2.28 (1.48)	1.53 (0.94)
Neurobehavioral							
Sleeping more than usual	3.00 (1.24)	3.33 (1.46)	2.81 (1.44)	2.52 (1.39)	2.49 (1.39)	2.15 (1.30)	2.00 (1.58)
Trouble sleeping	3.13 (1.46)	3.05 (1.94)	2.66 (1.52)	2.28 (1.57)	2.48 (1.47)	2.39 (1.48)	2.13 (1.54)
Irritability	3.00 (1.49)	2.90 (1.59)	2.43 (1.36)	1.85 (1.03)	2.07 (1.28)	2.09 (1.43)	2.21 (1.61)
Sadness	2.76 (1.48)	2.73 (1.44)	2.57 (1.50)	1.67 (0.91)	2.32 (1.70)	1.92 (1.12)	1.55 (0.82)

Means and SDs in the table are based only on those subjects who reported each symptom, i.e., subjects who did not report that symptom on that day are not included. Number of subjects assessed by day for pooled time points: day 3 *n* = 289, day 4 *n* = 36, day 5 *n* = 269, day 6 *n* = 45, day 7 *n* = 252, day 8 *n* = 38, and day 9 *n* = 10. Numbers do not add to total since some subjects contribute more than once to a pooled time point

^a^ *n* number of subjects assessed at each time point

Both the prevalence of symptoms (Fig. 1) and their severity (Table 6) declined over time, with two notable exceptions. First, a subgroup of somatic symptoms (fatigue, drowsiness, sensitivity to light, and sensitivity to noise; Fig. 1b) exhibited an increase in prevalence from time of injury to post-competition and/or post-competition to day 1. This was in contrast to the other somatic symptoms, which declined over the same period (Fig. 1a). Second, most neurobehavioral symptoms were more prevalent at post-competition and day 1 than at time of injury (Fig. 1d). This may reflect the additional time required for behavioral symptoms (such as sleep disturbances) to become evident.

The median time to resolution of all symptoms was 3 days. In 86 % of cases, symptoms resolved in 1 week or less (*n* = 304). Even though symptom prevalence and severity declined over time, some individuals developed new symptomology during the course of recovery. Additional symptom(s) developed after the time of injury in 18 % of cases (*n* = 66). In half of these cases, the additional symptoms developed within 4 h of the injury (*n* = 33), but 29 % developed additional symptoms at day 1 or later (*n* = 19).

## Discussion

Despite the significance of concussion as a public health problem, the epidemiology of concussion is not well-described. This paper reports data from a prospective study specifically designed to study concussion and provides detailed information on the incidence and characteristics of these injuries.

### Incidence

The concussion rates observed in this study are generally similar to those reported in studies from the United States (Clay et al. 2013). The incidence of sports-related concussion was approximately similar in the high school and the college settings in this study. However, the public health burden of concussion is far greater in the high school setting, since there are far more high school athletes (7.1 million) than collegiate athletes (380,000) (National Federation of State High School Association 2014; National Collegiate Athletic Association 2014).

### Concussion history

The observed association between history of previous concussion and incident concussion has also been observed in other studies (Guskiewicz et al. 2003; Schulz et al. 2004; Zemper 2003). At least two explanations are possible. First, repeated concussion may lower the biomechanical (acceleration/deceleration) threshold for subsequent concussions in an individual, possibly because neurons may become more vulnerable to injury due to decreased neurotransmitter activity along previously injured pathways (Nilsson et al. 1977; Giza and Hovda 2001; Slemmer et al. 2002). This is supported by the clinical observation that athletes who sustain a large number of concussions sometimes develop a chronically comprised threshold for concussion (e.g., “punch-drunk” boxers). However, there is conflicting evidence about whether cumulative neurocognitive degradation is linked to clinical head trauma, repetitive subconcussive impact, or neither (Matser et al. 1999; Collins et al. 2002; Iverson et al. 2004; Moser et al. 2005; Iverson et al. 2006; Broglio et al. 2006). Secondly, and perhaps more plausibly, the risk of concussion may be associated with concussion history for behavioral reasons. For example, some athletes experience a higher intensity of impacts to the head (in terms of the number, location, and force of collision), possibly because of the style of play of the team and/or individual. It is currently unclear whether risk of repeat concussion is due to a lowered threshold of vulnerability to subsequent trauma, behavioral factors such as style of play, or some combination of these factors. This issue demands further study because repeat concussion has been linked to early loss of neurological function (Guskiewicz et al. 2005) and clinical depression (Guskiewicz et al. 2007).

### Signs and symptoms

Sports teams, which often have ready access to health professionals trained in sports medicine, provide a “natural laboratory” for describing the signs and symptoms of concussion. These and previous results (McCrea et al. 2003) demonstrate that concussions present with a diverse range of symptoms, most of which gradually resolve within a week. Consistent with other studies of mild head injury (McCrea et al. 2003; Evans 1992; Powell et al. 1996; Haboubi et al. 2001), there was no single symptom that was universally present in all subjects.

Loss of consciousness and amnesias are dramatic signs of concussion which have historically been regarded as important markers of severity (Kelly and Rosenberg 1998). This study and others (McCrea et al. 2003) demonstrate that most concussions involve neither loss of consciousness nor amnesia. The CDC has launched a series of public awareness campaigns aimed at educating athletes, parents, coaches, and healthcare providers about how to identify sports-related concussions (National Center for Injury Prevention and Control (NCIPC) 2014).

### Prevention

In both football and soccer, the most frequent injury mechanism was a collision between two players. Strengthening or introducing rules that limit the potential for player collisions, and other uncontrolled high-energy contacts, may lower the incidence of concussion. A soccer example would be the introduction of a “fair head” rule, which would prohibit a player from contesting another player for the opportunity to head a ball when the second player had clear possession of the best position to receive the ball. In football, there may be scope for improved helmet design and fit to reduce the risk of concussion. Although helmets were originally designed to prevent severe head trauma (Daneshvar et al. 2011) rather than concussion, recent research has suggested that some helmets may out-perform others in preventing concussion (Rowson and Duma 2011). Enforcement of rules that limit overly aggressive head contact in games and practices is also important.

The incidence of concussion in women’s lacrosse (which prohibits contact) was similar to men’s lacrosse (which allows contact). Addressing stick-to-head contacts needs to be a priority for women’s lacrosse. It was also notable that goalkeepers in soccer had a higher incidence of concussion than goaltenders in lacrosse. This may reflect the fact that soccer goalkeepers dive to block the ball (potentially exposing them to contact from opponents feet and legs) and/or the use of protective helmets by goalkeepers in men’s and women’s lacrosse. Contact with a ball was a frequent mechanism of injury in soccer; however, we are unable to state what proportion were attempts to head the ball and what proportion were completely unintentional ball contacts to the head.

### Limitations

This study presents the large case series of concussions; however, some limitations are present in this study. These include the potential that some subjects experienced a concussion but did not seek care or disclose symptoms to the certified athletic trainer(s). Additionally, missing data at some assessment points create the potential for bias in our symptoms over time. We did not select a random sample from potential high schools and colleges, and our exposure data come from a convenience sample; therefore, representativeness cannot be guaranteed. Recall effects could have biased the observed association between previous concussion and incidence concussion, for example, if athletes with a prior history of concussion were more vigilant in seeking health care following head impact.

## Conclusions

The results from this study, one of the largest series of concussions on which detailed data have been assembled to date, underscore the fact that concussion symptomology is very diverse between individuals. In the sports studied here, the rate of concussion was similar between the high school and college settings and was much higher in games than in practices. Although football presents the highest risk for the athlete, the incidence in soccer and lacrosse is still significant. Our study further substantiates an apparent increased vulnerability to future concussions once an athlete has sustained an initial concussion. Research is needed to better understand the nature of this association.
